# Bright Light Therapy in Parkinson's Disease: An Overview of the Background and Evidence

**DOI:** 10.1155/2012/767105

**Published:** 2012-12-23

**Authors:** Sonja Rutten, Chris Vriend, Odile A. van den Heuvel, Jan H. Smit, Henk W. Berendse, Ysbrand D. van der Werf

**Affiliations:** ^1^Department of Psychiatry, VU University Medical Center, 1007 MB Amsterdam, The Netherlands; ^2^Department of Anatomy and Neuroscience, VU University Medical Center, Van der Boechorststraat 7, 1081 BT Amsterdam, The Netherlands; ^3^Department of Neurology, VU University Medical Center, 1007 MB Amsterdam, The Netherlands; ^4^Department of Sleep and Cognition, Netherlands Institute for Neuroscience, Royal Netherlands Academy of Arts and Sciences, Meibergdreef 47, 1105 BA Amsterdam, The Netherlands

## Abstract

Sleep disorders are common in Parkinson's disease (PD) and seem to be strongly associated with depression. It has been suggested that sleep disorders as well as depression are caused by a disturbed circadian rhythm. Indeed, PD patients are prone to misalignment of their circadian rhythm due to various factors, and many patients with PD display a phase advance of their circadian rhythm. Current treatment options for sleep disorders and depression in patients with PD are limited and can have serious side effects; alternative treatments are therefore badly needed. Bright light therapy (BLT) restores circadian rhythmicity effectively in mood- and sleep-disturbed patients without PD. The few studies that focused on the efficacy of BLT in patients with PD demonstrated a positive effect of BLT not only on sleep and mood but also on motor function. More research on the neurobiology and efficacy of BLT in PD is warranted.

## 1. Introduction

In addition to the characteristic motor symptoms, patients with Parkinson's disease (PD) experience many nonmotor symptoms, comprising a variety of cognitive, autonomic, sensory, neuropsychiatric, and sleep disturbances [1, 2]. Sleep disturbances and disorders (as defined in Table 1) including reduced total sleep time, reduced sleep efficiency, increased sleep fragmentation, rapid eye movement (REM) sleep behaviour disorder, and excessive daytime sleepiness, occur in about 60–95% of PD patients [3–6]. Sleep influences motor symptoms. The so-called “sleep benefit”, an improvement of motor functions upon awakening that occurs in more than 40% of PD patients, is attributed to improved dopaminergic function as a result of increased storage of dopamine in nigrostriatal terminals during sleep [7]. Moreover, melatonin, a hormone secreted by the pineal gland at night, has been suggested to worsen motor symptoms in PD patients [8]. 

Sleep disorders in PD often coincide with depression [6]. Depression occurs in 35–50% of patients throughout the course of the disease [9, 10]. It has a major impact on overall functioning of PD patients: depressed PD patients score lower on scales assessing activities of daily living and exhibit more cognitive problems [9, 11, 12].

Sleep disorders and depression are two of the most important factors influencing quality of life of PD patients and their caregivers [4, 9, 13, 14]. Unfortunately, treatment options are limited, and adding pharmacological agents raises nonadherence in PD patients [15]. Moreover, medication can induce serious side effects in PD patients. Hypnotic drugs, often prescribed for sleep disorders, worsen daytime sedation and the risk of falling and are therefore less suitable for PD patients [16]. Melatonin might ameliorate subjective sleep disturbances in PD patients, but objective improvement of sleep quality is minimal [17, 18]. Since a number of studies indicate that melatonin has unfavorable motor effects through interaction with dopamine pathways, more research is warranted on the effects of exogenous melatonin in PD patients [19–22].

Tricyclic antidepressants (TCAs), used in the treatment of depression in PD, can cause orthostatic hypotension, sedation, cognitive and anticholinergic adverse effects, in addition to extrapyramidal adverse effects, that may potentially worsen motor symptoms [23, 24]. Results of studies focussing on the tolerability of selective serotonin reuptake inhibitors (SSRIs) are inconclusive [23–25]. Levodopa treatment can alleviate nocturnal akinesia and thus improve sleep but can conversely negatively influence sleep by reducing the duration of REM sleep and increasing REM sleep latency [26]. Anticholinergics and dopamine agonists increase the risk of nighttime hallucinations [27]. The latter are also associated with sudden attacks of daytime sleepiness [26], which may hamper quantity and quality of nighttime sleep. Behavioral and psychotherapeutic interventions are often less feasible due to cognitive dysfunction and dementia [28]. It is evident that there is a great need for an effective and patient-friendly alternative for treating sleep disorders and depression in PD patients. 

Sleep problems and depressive symptoms often cooccur in PD [6, 14]. Dysfunction of the biological clock might be a common underlying causal factor for these disorders, providing a promising potential target for treatment [29, 30]. Bright light therapy (BLT) restores circadian rhythmicity and therefore effectively treats affective disorders and insomnia, and increases sleep efficiency [31–38]. Additionally, it might lead to improvement of motor symptoms in PD [8, 39, 40]. BLT has few contraindications and side effects and may therefore be an elegant alternative for the treatment of PD-related depression and sleep disturbances.

This paper gives an overview of the neurobiology of the biological clock and the factors that contribute to its desynchronization in PD. Furthermore, we review the evidence for BLT as a treatment for sleep disorders, depression and motor symptoms in patients with PD, and provide recommendations for administration of BLT. 

## 2. The Circadian Rhythm and Consequences of Desynchronization

To understand the effects of BLT, one needs to understand the (patho)physiology of circadian rhythmicity, as explained in this section. The circadian rhythm is generated by the circadian pacemaker, a group of about 10,000 neurons located in the suprachiasmatic nucleus (SCN) of the hypothalamus. Its endogenous rhythm is slightly different from the 24-hour day-night cycle and has to be entrained by signals (or “zeitgebers”) such as light, activity, and food [41]. Light excites specialized melanopsin containing ganglion cells in the retina, that project a “daytime” signal towards the SCN via the retinohypothalamic tract [42]. The output signals of the SCN convey circadian timing information to brain areas regulating behavior, body temperature, autonomic and neuroendocrine systems, including the secretion of melatonin by the pineal gland [42]. The secretion of melatonin is inhibited by the SCN during the light cycle, but the SCN also contains melatonin receptors that inhibit SCN firing, thereby creating a negative feedback loop [43, 44]. 

Desynchronization of the biological clock can be caused by a variety of factors that influence the input of the SCN [45]. A disturbed circadian rhythm is probably a major common causal factor in both depression and sleep problems [29, 46, 47]. Some of the major neurotransmitters implicated in mood regulation, including serotonin, norepinephrine, and dopamine, as well as their receptors, show a circadian rhythm in their levels, release, and activity [48]. Various polymorphic variations of clock genes such as TIM, BMAL1, and PER2 are associated with mood disorders [29]. Research on the diurnal variability of mood has shown that misalignment of the circadian rhythm can induce mood changes [47]. Some patients with a depressive disorder display a phase advance in circadian rhythm, as exhibited by a shift in melatonin and cortisol rhythms [36, 47]. Dysfunction of the circadian clock can lead to sleep fragmentation or insomnia [42, 45]. 

The interaction between sleep and depression likely comprises more than a failure of the biological clock. Insomnia or hypersomnia are well-known symptoms of depression, but sleep disturbances can cause depressive symptoms as well [29, 47, 49–55]. Emotional hyperarousal may increase autonomic activity, resulting in sleep difficulties [53]. This is confirmed by the fact that depressed patients show altered sleep architecture, which normalizes after successful treatment [50]. On the other hand, emotionality is frequently negatively toned in insomnia and poor sleep [47, 51], and studies on sleep deprivation showed enhanced emotional physiological responses to negative stimuli [49, 52, 55]. During REM sleep, emotional intensity of previous affective experiences is decreased [54, 56, 57]. Functional Magnetic Resonance Imaging (fMRI) studies show that sleep deprivation leads to increased activation of the amygdala in response to negative aversive stimuli [58–60]. These findings strongly suggest that sleep is relevant for maintaining adaptive emotional regulation and reactivity [29, 54]. 

In short, sleep disturbances and depression seem to be highly correlated. A disturbed biological rhythm might be a common underlying factor and therefore an important starting point for treatment. However, the directionality of the relationship between these three remains uncertain, and more research on this subject is warranted. 

## 3. Desynchronization of the Circadian Rhythm in Parkinson's Disease

PD patients are prone to desynchronization of their biological clock due to various factors that will be discussed in this section. The neurodegenerative process in PD leading to dopamine depletion is one of the underlying causes, since recent research links dopamine directly to the circadian rhythm [61–63]. Striatal dopamine metabolism seems to be regulated by clock proteins such as PER2 [62]. Reciprocally, stimulation of dopamine receptors affects the rhythm of expression of clock genes such as PER1 and PER2 in the striatum [61, 63]. Dopamine also regulates the rhythmic expression of melanopsin in retinal ganglion cells, thereby influencing the entrainment of the circadian rhythm by light [64].

In many patients with PD, factors hampering the SCN input contribute to desynchronization of the circadian rhythm. Firstly, exposure and sensitivity to zeitgebers decrease. Retinal illumination decreases in the elderly due to pupillary miosis and reduced crystalline lens light transmission, especially of short wavelengths [65]. This leads to partial light deprivation of the SCN and pineal gland. Additionally, PD patients, just like many elderly patients, may be more inclined to stay indoors due to motor problems or a decreased postural balance and expose themselves less to environmental light and physical activities [45]. Entrainment of the circadian rhythm is thwarted by a decreased exposure to zeitgebers. 

The amplitude of the circadian rhythm decreases in patients with PD, as reflected by a decrease in sympathetic activity during the day, diminishing of the diurnal variation of cortisol secretion, and a decrease of the amplitude of the melatonin secretion rhythm [19, 66, 67]. This flattening of circadian rhythms makes them more prone to desynchronization. 

Sleep in PD patients can be disrupted by both motor (e.g., nocturnal akinesia and dystonia) and nonmotor symptoms such as nocturia [1, 68]. Additionally, PD patients may experience periodic limb movement disorder, restless legs syndrome, REM sleep behavior disorder, and excessive daytime sleepiness [4, 26, 68], all contributing to a reduced quality and/or quantity of sleep. PD-related neuropsychiatric disorders such as benign hallucinations and psychosis can also disturb sleep [6]. Emotional stress, caused by having a progressive neurodegenerative disorder that increasingly results in disability, may interact with the basic homeostatic and circadian drives for sleep through the interaction between affect-related regions and regions that control sleep and wake [30]. A disturbed sleep-wake cycle results in conflicting SCN input. 

Finally, pharmacological treatment of PD with dopaminergic drugs also influences sleep/wakefulness mechanisms. Levodopa use can lead to a decrease of sympathetic activity during the day and disappearance of the sympathetic morning peak [69]. Levodopa influences sleep architecture, reducing the duration of REM sleep and increasing REM sleep latency [26]. 

**Figure 1:**
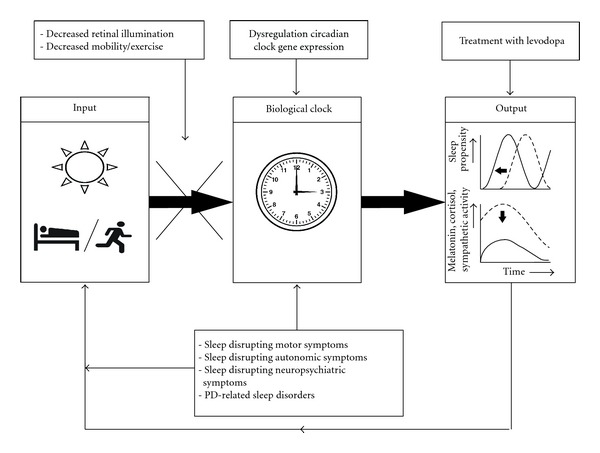
The input of the biological clock by zeitgebers is both decreased and conflicted due to various motor and non-motor symptoms in PD. Dopamine depletion due to PD disrupts circadian clock gene expression, and its treatment with levodopa influences both sleep structure and sympathetic activity. These factors all alter output of the biological clock: there is a phase advance and flattening of the circadian rhythm as displayed by hormone levels and sympathetic activity. In turn, the alteration of circadian rhythmicity has a negative influence on (input of) the biological clock, leading to a downward spiral resulting in sleep disturbances and depression.

All of the abovementioned factors may contribute to a desynchronization of the circadian rhythm in PD, as displayed in Figure 1. In several small studies, levodopa-treated PD patients display a phase-advanced circadian rhythm compared to healthy controls and de novo PD patients [19, 70, 71], making them vulnerable to depression and sleep disorders. Indeed, PD patients have more frequent awakenings at night and a reduced sleep efficiency compared to healthy controls [68]. BLT acts as a strong zeitgeber and may therefore restore circadian rhythmicity in PD patients.

## 4. Efficacy of Bright Light Therapy

In the last couple of years, research on the efficacy of BLT has shifted from adults to the elderly and specifically to PD patients. In 2005 a meta-analysis demonstrated that BLT is effective in treating seasonal affective disorder (SAD) and nonseasonal depression in adults, with effect sizes equivalent or superior to psychopharmacologic treatment [31]. BLT has few side effects and is therefore considered a patient-friendly treatment [32, 72]. 

Two recent large randomized controlled trials focused on the efficacy of BLT for nonseasonal depression in the elderly [32, 33]. Lieverse et al. stated that the positive effects of BLT were due to improved circadian rhythmicity, as displayed in their study by (1) an increased steepness of the evening rise of salivary melatonin levels, (2) a reduction of 24-hour urinary cortisol excretion, and (3) a trend-significant accelerated diurnal decline in salivary cortisol levels [32]. Riemersma-van der Lek et al. demonstrated that BLT attenuated cognitive and functional decline and positively influenced mood in 189 residents of group care facilities, of which 87% had dementia. In this study, BLT only improved sleep when it was combined with the administration of melatonin [33]. In other studies, BLT as monotherapy was effective in improving both sleep efficiency and quality and in reducing daytime sleepiness in elderly patients with and without dementia [32, 34, 35, 37]. 

To summarize, BLT seems to be effective in treating sleep disorders as well as depression. Most of the abovementioned studies, however, excluded patients with disorders such as PD. Only four studies have addressed the use of BLT in PD [8, 39, 40, 73]; these will be discussed in the following section. The first study in which BLT was used in PD patients is only available in Russian and is therefore not included in this overview [73]. 

Willis and Turner described a case series of 12 patients with PD and insomnia and/or depressive symptoms [40]. They used BLT of 1000–1500 Lux for 60 to 90 minutes prior to normal bedtime during two to five weeks. Of the eight participants that reported significant problems with falling asleep, seven showed improvement in the onset and continuity of sleep after BLT treatment. Most patients reported this effect within two to three days after commencing BLT, and this lasted for several days after discontinuation. Six of 11 patients showed a noticeable improvement of mood. The antidepressant effect lasted for several weeks, even after discontinuation of BLT, and was paralleled by increased socialization. BLT resulted in improved motor function in most PD patients, with the strongest effects on bradykinesia and rigidity. After BLT, dopamine replacement therapy was reduced to a level ranging from 13 to 100% in five subjects, while antidepressants and hypnotic drugs were reduced or eliminated in two patients. Younger patients, especially those that were medication naïve, responded better to BLT than those over 75 years of age, and adherent patients had a better therapeutic response than those who used it intermittently. 

In a RCT by Paus et al., 18 PD patients treated with BLT of 7500 Lux were compared to 18 PD patients receiving placebo light of 950 Lux [39]. Light was administered for 30 minutes in the morning during two weeks. PD-related symptoms were assessed with the Unified Parkinson's Disease Rating Scale (UPDRS); depression was measured with the Beck Depression Inventory (BDI). Patients who received BLT showed a significant improvement on UPDRS sections I (evaluation of mentation, behavior, and mood), II (self-evaluation of the activities of daily life), and IV (Hoehn and Yahr Scale) compared with the control group. Improvement of UPDRS I and II did not correlate with changes in BDI scores, implying that the effects of BLT on behavior and daily functioning were independent of changes in mood. There was no significant difference on UPDRS section III (clinician-scored motor evaluation), except for a slight attenuation of tremor. Regarding sleep, the only sleep parameter investigated was a one-item daytime sleepiness scale, which did not show a between-group difference. Mood improved significantly, but moderately, in the BLT group, as demonstrated by an average decrease of 2.2 points on the BDI. No significant improvement of BDI scores occurred in the control group. The short treatment duration and the fact that only mildly depressed patients were included might explain the modest effects of BLT on motor function and depression [49]. 

(Willis et al. 2012, [8]) performed a retrospective, open label study monitoring 129 levodopa-treated PD patients for a period ranging from a few months to eight years [49]. These patients were all prescribed BLT at a dose of 4000 to 6000 Lux for one hour prior to bedtime. Depending on the degree of adherence, PD patients were divided in the early quit group (EQUIT; patients that withdrew from BLT immediately after intake), the adherent group and the semiadherent group. Twelve patients suffering from other neurological conditions served as a control group. Motor function was assessed with three timed motor tests and a global rating scale. Psychiatric symptoms and sleep were evaluated on a global rating scale during an interview. Total drug burden (TDB) was determined and monitored over time. There was a slight deterioration of insomnia seen in EQUIT patients, while adherent patients showed an acute and dramatic improvement. Adherent patients displayed a significant improvement of bradykinesia, rigidity, balance, and motor tests, while the motor parameters in the EQUIT group deteriorated over time. In the semiadherent group, these parameters varied over time and appeared associated with periods of nonadherence with BLT or changes in drug regimen. All groups displayed an improvement of depression over time, with the most robust improvement seen in adherent patients. Anxiety did not change in the EQUIT group in contrast to other groups, with the greatest improvement in the adherent group. The adherent and semiadherent groups required an increase in TDB in, respectively, 13 and 15% of cases, while 91% in the EQUIT group required increased medication. Moreover, patients in the EQUIT group were on similar doses of dopamine replacement therapy as the other PD patients but displayed more severe PD than those who received BLT. In the adherent group, morbidity improved over the course of years, while in the EQUIT group, progression of PD severity was as expected. Limitations of this study are the fact that the study was not blinded or placebo controlled, and that patients were monitored for different periods of time. 

Depression can lead to psychomotor retardation [74], so the improvement of motor function in these studies could be attributed to a decrease in depressive symptoms. However, in the study by Paus et al (2007). there was no improvement of motor function in the subjects that demonstrated a significant decrease of the BDI score [39]. More likely, the positive effects of BLT on motor symptoms in PD result from a restored balance between melatonin and dopamine [8]. A number of studies indicate that melatonin has unfavorable motor effects through interaction with dopamine pathways [19–22]. 

 Another point that is not addressed in these studies is the effect of other zeitgebers on the improvement in circadian rhythmicity. All subjects were given BLT at a set time, prior to bedtime or after awakening in the morning. This may have improved the daily rhythm and sleep-wake cycle of participating patients. Behavioral and psychological interventions are also effective in treating insomnia [75, 76]. 

Taken together, these studies display a positive effect of BLT on mood, sleep, and motor functions in PD patients. However, since these studies were relatively small and suboptimally designed, further studies on the efficacy of BLT in treating both nonmotor motor and motor symptoms in patients with PD are warranted. 

## 5. Recommendations for Administration of BLT

In this section, recommendations for administration of BLT as well as information on contraindications and adverse effects are provided. Due to a lack of research on BLT in PD patients, the majority of these recommendations are based on research in patients without PD, so we must stress that (adverse) effects in PD patients might be different. More research on the effects of BLT in PD patients needs to be done before BLT can be used for PD patients in daily clinical practice.

There are many types of light boxes available. Clinically tested models yield a maximum illuminance of 10.000 Lux at a comfortable sitting distance of about 30 cm [38, 77]. At this intensity, a duration of 30 minutes per session is usually sufficient, while lower intensities require longer sessions [31, 78, 79]. It is advisable to use a light box with a complete ultraviolet (UV) filter, since cumulative UV radiation can be harmful to eyes and skin [80, 81].

Time of administration of BLT depends on the nature of the patient's complaints and his or her individual chronotype. Morning light advances the biological clock and has proven to be effective in treatment of depression [77, 78]. However, patients with PD probably have a phase-advanced circadian rhythm, and one might argue that evening BLT might be more efficient [8, 19, 40, 70, 71]. On the other hand, Paus et al. (2007) demonstrated that morning light can improve mood and sleep in PD patients as well [39]. BLT is most effective when administered relative to individual chronotype [38, 82]. The chronotype can be assessed with the Morningness-Eveningness Questionnaire (MEQ), which correlates with the time of onset of evening rise in melatonin secretion and circadian variation of oral temperature [78, 82, 83]. An online version of the MEQ at the website of the Center for Environmental Therapeutics (http://www.cet.org/) contains a table of the recommended timing of morning BLT based on the MEQ-score. Strict adherence to BLT is necessary to maximize efficacy [8].

There is no consensus on the total duration of treatment with BLT in nonseasonal depression or insomnia. In PD patients, followup after discontinuation of BLT was only performed in the study of Willis and Turner (2007) [40]. They observed that the antidepressant effect of BLT lasted after discontinuation of therapy, but sleep deteriorated after a couple of days. These findings correspond with a large study on the effects of BLT in the elderly patients with a nonseasonal depression [32]. Since it might take months before BLT can exert positive effects on motor function, a long treatment duration of PD patients might be necessary [8]. More research on both timing and duration of BLT in patients with PD is warranted.

Cumulative light energy can cause damage to skin and eye tissues, especially short-wavelength UV light [78, 80, 81]. Patients with porfyria, macular degeneration, retinal dystrophy, lupus erythematosus, chronic actinic dermatitis, and solar urticaria can have photosensitization reactions to light and should only receive BLT under monitoring of an ophthalmologist or dermatologist [78]. Moreover, some pharmacological agents are known to photosensitize the skin or retina, including some of the tricyclic antidepressants, tetracyclic antibiotics, and antiarrhythmic drugs [78]. These medications should be stopped before commencing BLT.

In a study of 70 subjects receiving BLT for a SAD the most often reported adverse effects were headache, eye or vision problems and nausea [72]. No oculoretinal changes were detected during ophthalmologic evaluations of patients receiving treatment with BLT to up to six years [84]. Some cases of BLT-induced (hypo)mania have been described, requiring discontinuation of BLT and medication [72, 85, 86]. However, in patients with a known or suspected bipolar disorder BLT can be administered when the patient is using a mood stabilizer [80]. Nevertheless, side effects of BLT are mostly mild and usually resolve within a couple of days [32, 33, 72].

## 6. Conclusion

Sleep disturbances are common in PD and are strongly associated with depression [3–6]. A disturbed circadian rhythm may be a common underlying factor in both disorders [29, 46, 47]. PD patients are prone to misalignment of the circadian rhythm due to dopamine deficiency as well as various other factors that disrupt input to the SCN [1, 4, 26, 45, 63, 66, 68]. Indeed, many patients with PD display a phase advance of their circadian rhythm [19, 70, 71], which may contribute to the increased prevalence of sleep disturbances and depression [6, 9, 10]. 

Since the current treatment options for sleep disturbances and depression in PD are limited and can have serious side effects [16, 23], alternative treatments are badly needed. BLT restores circadian rhythmicity and is an effective treatment for depressive disorders and insomnia in the general population [31–35, 37]. So far, little research has focused on the efficacy of BLT in patients with PD [8, 39, 40, 73]. The studies that have been performed were small and suboptimally designed yet demonstrated a positive effect of BLT on sleep and mood in patients with PD. Moreover, BLT may positively influence motor function, possibly through a restored balance between melatonin and dopamine [8, 40]. It might thus facilitate a dose reduction of dopaminergic medication [8, 40]. BLT has few side-effects and is therefore patient friendly [32, 72]. Nevertheless, more research is warranted to demonstrate the efficacy and underlying mechanism of BLT in PD.

## Figures and Tables

**Table 1:** Definitions of sleep terminology.

Term	Definition
Sleep disturbance	Sleep pattern divergent of what is considered to be normal as objectively measured, for example, by polysomnography.
Sleep disorder	Medical disorder involving sleep, resulting in suffering or reduced functioning, including dyssomnias and parasomnias.
Sleep onset latency	Time interval between time of turning of the lights and onset of sleep.
Sleep efficiency	Ratio of the time spent asleep to the amount of time spent in bed.
Chronotype	Individual internal timing type regarding preferred time for mental and physical activity and sleep.
Homeostatic sleep drive	Drive to sleep that gradually increases with prolonged wakefulness and decreases during sleep.
Sleep fragmentation	Disrupted sleep cycle due to interruption of a sleep stage, as a result of the appearance of a lighter sleep stage or wakefulness.
Sleep phase advance	Forward shift of the sleep/wake rhythm, as demonstrated by the time of the nocturnal elevation of plasma melatonin.
Insomnia	Sleep disorder comprising difficulty initiating and/or maintaining sleep or nonrestorative sleep for at least one month, resulting in significant distress and/or impaired daytime functioning
REM sleep behavior disorder	Parasomnia characterized by “acting out” of dreams during REM sleep due to absence of normally occurring muscle atonia.
Excessive daytime sleepiness	Parasomnia characterized by excessive sleepiness during the day, often with hypersomnia and the occurrence of sleep attacks.
Periodic limb movement disorder	Sleep disorder characterized by involuntary limb movements causing fragmented sleep.
Restless legs syndrome	Syndrome characterized by unpleasant sensations in one or more limbs, exacerbated by rest and relieved with activity, paired with a strong urge to move the affected limbs, often with paresthesias or dysesthesias.
